# An exploration of patients’ expectation of and satisfaction with surgical outcome

**DOI:** 10.1007/s00586-013-2971-6

**Published:** 2013-08-30

**Authors:** Alison H. McGregor, Caroline J. Doré, Tim P. Morris

**Affiliations:** 1 Surgery and Cancer, Faculty of Medicine, Imperial College London, Charing Cross Hospital Campus, London, W6 8RP UK; 2 MRC Clinical Trials Unit, Aviation House, 125 Kingsway, London, WC2B 6NH UK

**Keywords:** Spinal surgery, Satisfaction, Expectations, Outcome

## Abstract

**Purpose:**

The majority of studies of surgical outcome focus on measures of function and pain. Increasingly, however, the desire to include domains such as patients’ satisfaction and expectations had led to the development of simple measures and their inclusion into clinical studies. The purpose of this study was to determine patients’ pre-operative expectations of and post-operative satisfaction with the outcome of their spinal surgery.

**Methods:**

As part of the FASTER randomised controlled trial, patients were asked pre-operatively to quantify their expected improvement in pain and health status at 6 weeks, 6 and 12 months following surgery using 100 mm visual analogue scales (VAS), and to indicate their confidence in achieving this result and also the importance of this recovery to them. Patients were then asked to rate their satisfaction with the improvement achieved at each post-operative review using 100 mm VAS.

**Results:**

Although differences between patients’ expectation and achievement were minimal 6 weeks post-operatively, there was a clear discrepancy at 6 months and 1 year, with patient expectations far exceeding achievement. There were significant correlations between failure to achieve expectations and the importance patients attached to this recovery at each post-operative assessment, but not with their confidence in achieving this result. Satisfaction levels remained high despite expectations not being met, with discectomy patients being more satisfied than decompression patients.

**Conclusions:**

Patients’ pre-operative expectations of surgical outcome exceed their long-term achievement. The more importance the patient attached to a good outcome, the larger is the discrepancy between expectation and achievement. Despite this, satisfaction levels remained high. The impact of unrealistic expectations on outcome remains unclear.

## Background

Over the past decade there has been a growing emphasis on evaluating the patient’s perspective, particularly in spinal surgery, leading to the proposal of five core domains to record outcome: specific back function, generic health status, pain, work disability and patient satisfaction [3]. This paper focuses on the exploration of patient satisfaction. Satisfaction is a broad term and in relation to assessing outcome, it has been described as a multi-dimensional measure that encompasses a range of issues including the patient’s belief in what the treatment can provide, expectations of what they want the treatment to achieve, the level of pre-treatment symptoms and the relative change in these symptoms, as well as the process and delivery of the treatment which can include environment, location and staff issues [2, 10].

Thus, it is important to consider what is meant by the term ‘patient satisfaction’ and what part of the care process or outcome it pertains to. A range of approaches have been used in an attempt to address this including global assessment of satisfaction on Likert scales [37, 38], the use of visual analogue scales (VAS) to assess global satisfaction and satisfaction with key outcomes [12, 26, 33], the use of global multi-dimensional scales such as the client satisfaction questionnaire and the patient satisfaction questionnaire [14], and disease-specific questionnaires such as the patient satisfaction scale [14]. As yet there is no clear consensus on the best approach, making interpretation of findings complex. Also, other factors are known to influence satisfaction, particularly socio-demographic factors. This is eloquently described by Carr-Hill [4], who uses the example of satisfaction with the NHS in the UK varying considerably between the older population, who can recall health care prior to the establishment of the NHS, and the younger population, who have always had access to the NHS.

Measures of satisfaction have increasingly been accompanied by measures of expectations. A recent study of patient satisfaction with joint arthroplasty suggested that overall satisfaction can be based on three facets: meeting pre-operative expectations, achieving satisfactory pain relief following surgery and hospital experience [11]. This association between treatment outcome and patient’s pre-treatment expectation has previously been noted [4, 13, 26, 30], with expectation defined as how a patient thinks they will function following surgery [15]. There appears to be strong evidence that positive expectations are associated with positive outcomes [15, 19, 30, 34], but clearly more work is needed to understand these complex relationships as some patients can describe high levels of satisfaction, but report a poor outcome.

Therefore as part of a clinical trial exploring the value of rehabilitation and/or educational material in the post-operative management of spinal surgery (FASTER study—function after spinal treatment, exercise and rehabilitation), this study sought to establish patients’ pre-operative expectations of, and satisfaction with, the outcome of surgery in the short and longer term. Both expectation and satisfaction were explored in terms of pain and quality of life. The influence of the underlying disease process and subsequent post-operative management on satisfaction was also explored.

## Methods

### Trial design

The FASTER trial was designed to determine the optimal post-operative management of spinal surgery patients investigating the possible benefits of a rehabilitation programme and/or an educational booklet. It was a multi-centre, parallel group, factorial, randomised controlled trial approved by the local research ethics committee. The full details and primary outcomes have been reported previously [23, 24, 27]. In summary, the study recruited patients who were scheduled for primary surgery for either a lumbar decompression or discectomy procedure. Patients recruited and consented into the study were randomised with stratification by surgeon and surgical procedure using random permuted blocks and a 2 × 2 factorial design to receive:

- Factor 1—either a 6-week programme of post-operative rehabilitation or the relevant surgeon’s usual post-operative care.
- Factor 2—either an educational booklet (“Your Back Operation” see below) or the surgeon’s usual post-operative advice.

**Fig. 1:**
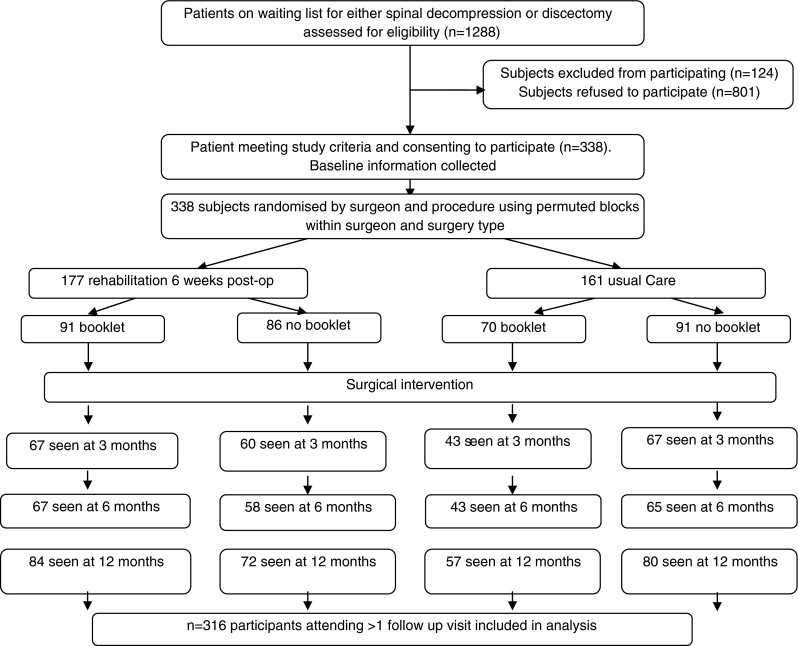
Consort diagram of patients progression through the study

This created four study groups: rehabilitation-only, booklet-only, rehabilitation-plus-booklet, and usual care-only (Fig. 1). Patients were assessed using a range of validated outcome measures pre-operatively and then at 6 weeks, 3, 6, 9 and 12 months post-operatively. Only the data generated pre-operatively and at 6 weeks, 6 and 12 months were used in this analysis since the additional assessments were to facilitate economic analysis and did not collect patient satisfaction or achievement. Surgeons were blinded to the data generated by the questionnaires since none included information routinely collected.

### Study population

Twenty surgeons participated in this study (8 orthopaedic and 12 neurosurgical) across seven different hospital sites in the London region. Patients were recruited by the trial coordinator and written informed consent obtained. Patients were approached if they were awaiting primary spinal surgery and presented with signs, symptoms and radiological evidence of either (a) lateral nerve root compression or (b) lumbar disc prolapse. The surgery was performed according to the surgeon’s routine practice for that condition (i.e. either lateral or central root canal decompression or discectomy) and the details were recorded. Similarly, patients were informed and consented for their surgery in accordance with their surgeon’s usual approach. Surgical approaches were not standardised because we aimed to reflect clinical practice, thereby increasing the generalisability of our results. Patients were excluded if they presented with any condition where either the intervention or the rehabilitation might have an adverse effect on the individual: previous spinal surgery; spinal surgery where a fusion procedure was planned due to the unknown hazards of the rehabilitation programme for this type of surgery; pregnant women; inadequate ability to complete the trial assessment forms; unable to attend or unsuitable for rehabilitation classes.

### Trial interventions

The interventions are briefly outlined below, but further details have been previously reported [23].

Rehabilitation programme: those patients allocated to the rehabilitation arms were invited to start the programme 6–8 weeks post-operatively. The rehabilitation programme consisted of 12 1-h classes led by an experienced physiotherapist.

Educational booklet: on discharge from hospital patients randomised to the booklet arms received a copy of “Your Back operation” [35].

Usual care: those allocated to usual care were managed according to their surgeon’s usual practice which was often limited to advice and a post-operative follow-up at 6–12 weeks.

### Outcome measures

Patient assessed outcomes were: 100 mm VAS, to record average back and leg pain as per Jensen et al. [16], and EQ-5D a standardised tool which measures health outcome (http://www.euroqol.org/eq-5d/what-is-eq-5d.html) used to assess overall health state [1, 8]. The assessment of expectation and satisfaction is outlined below:

#### Expectations

Pre-operatively, patients were asked to rate what they expected in a range of variables at the key outcome points (6 weeks, 6 months and 1 year post-surgery) using a 100 mm VAS [26]. These variables included their state of health (referred to hereafter as VAS health) and their levels of back and leg pain (with 0 representing very poor health state or no pain and 100 excellent or worst pain possible, respectively). For example in relation to leg pain, they were asked “how much leg pain do you think you will have a year after your surgery?” In addition they were asked to rate how important it was to them to achieve this level of recovery in their state of health, and how confident they were of achieving this recovery. Again this was assessed on 100 mm VAS ranging from not at all important to very important and not at all confident to very confident, respectively. In addition at the final review at 1 year, patients were asked to rate if the surgery had achieved what they had expected it to achieve, with the 100 mm scale ranging from ‘not at all’ to ‘definitely’.

#### Satisfaction

At each of the post-operative reviews, patients were asked to rate their satisfaction with their improvement since surgery on a 100 mm VAS ranging from totally dissatisfied to very satisfied with respect to health status, back pain and leg pain. For example, in relation to leg pain they were simply asked “how satisfied are you with the improvement in your leg pain?”

This approach to measuring expectation and satisfaction has been used previously [18, 26], but has not been fully validated.

### Statistical methods

The power calculation for the clinical trial has been previously reported [23]. This paper explores three key variables: VAS of health, leg pain and back pain. Conventionally, higher values of VAS health score indicate a good outcome, whilst higher values of VAS leg and back pain indicate a high pain score. The scales for leg and back pain have therefore been reversed for all analyses presented here such that a high score always indicates a good outcome (i.e. low pain), to give them the same direction of interpretation as VAS health. Note that satisfaction with outcome has the same interpretation whichever variable is being referred to, i.e. a higher score represents a higher level of satisfaction.

There was skew in the marginal distributions of expected outcomes and large skew in the marginal distributions of confidence and importance of achieving expectations of overall health, and medians are used to describe and summarise these variables. Rank correlations were therefore used to correlate expectation with each of achievement, confidence and importance.

We used mixed regression models [28] to investigate mean change over time in expectation, achievement and satisfaction, which takes into account the mixture of between- and within-patient information. (Despite the skew in the marginal distribution of expected outcomes, this disappeared from the residuals of the mixed models.) These models included random effects for patients (to allow for the dependence between measurements on each patient at different time points), fixed effects for follow-up time points (as we were interested in these specific time points) and were adjusted for baseline value of outcome (to adjust for the variability between patients in their initial status). An unstructured variance–covariance matrix was used (i.e. correlations between any two time points were allowed to differ).

We compared the effect of the two (randomised) trial interventions and of the (non-random) surgical procedure on satisfaction with outcome using mixed regression models. These used random effects for patients and surgeons, fixed effects for surgical procedure, booklet and rehabilitation, and adjustment for baseline value of the outcome. An unstructured variance–covariance matrix was used. For these analyses, non-trivial amounts of outcome data—up to 29 %—were missing. Our treatment effect estimates are based on mixed models using all available data. Such models correctly account for missing data uncertainty, assuming outcomes are ‘missing at random’ given the observed data in the model [31]. However, to make the missing at random assumption more plausible, we used multiple imputation including extra observed (‘auxiliary’) information from other variables [32], including ODI, leg pain, back pain, VAS overall health rating, anxiety and depression. We used multiple imputations by chained equations [36], running ten cycles before storing the imputed values and creating 30 imputed datasets in total. Results were combined in the standard way using Rubin’s rules [31].

## Results

The detailed characteristics of this population have been previously presented [24]. In summary, a total of 1,288 patients were approached to take part in this study which ran between June 2005 and March 2009; of these, 124 did not meet the inclusion criteria and 338 were enrolled to the study. This resulted in 91 patients randomised to receive rehabilitation and the booklet, 86 to receive rehabilitation only, 70 to receive only a booklet and 91 to receive normal care. The Consort diagram documenting patient’s progress through the stages of the study has been plotted in depth, but an overview is presented in Fig. 1; further details including study demographics can be found in McGregor et al. [24].

### Analysis of patients’ expectations

In the first part of the analysis, we correlated patients’ outcomes achieved following surgery with what patients had expected before surgery. There is only a moderate correlation *p* < 0.05 (explaining approximately 4–8 % of the variation seen) between the outcome patients achieved and what they expected, and this correlation was similar for both short- and longer-term time points (Table 1).

**Table 1:** Rank correlations (*p* value) between expectation and achievement at 6 weeks, 6 and 12 months post-operatively

Variable	6 weeks	6 months	12 months
VAS health	0.26 (<0.001)	0.20 (0.003)	0.20 (<0.001)
Leg pain	0.25 (<0.001)	0.28 (<0.001)	0.20 (<0.001)
Back pain	0.20 (0.006)	0.22 (0.001)	0.21 (<0.001)

**Fig. 2:**
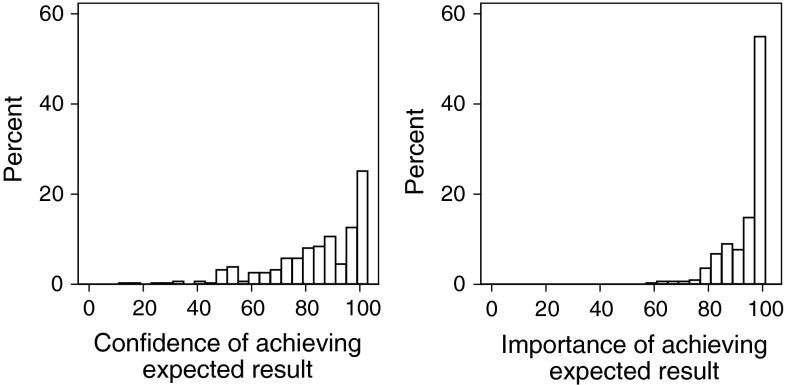
Histogram of patients’ confidence in outcome and how they ranked the importance of this (0 = low confidence/low importance; 100 = extremely confident/important respectively)

Not surprisingly, as shown in Fig. 2, pre-operatively most patients were confident that they would achieve their expected improvement in their health and it was very important to the majority of patients to achieve these expectations.

The difference between expectation and achievement was calculated by subtracting the outcome achieved from the expectation recorded by the patient pre-operatively. The results are presented in Table 2 and suggest that while differences between expectations and outcome achieved were negligible at 6 weeks, patients expected a better health status at 6 and 12 months than that achieved.

**Table 2:** Difference between patients’ pre-operative expectation and outcome achieved post-operatively [median difference (95 % CI); *p* value]

Variable	6 weeks	6 months	12 months
VAS health	1 (−4 to 5); 0.18	8 (5–10); <0.0001	14 (10–17); <0.0001
Leg pain	2 (−1 to 6); 0.24	1 (0–5); 0.0001	13 (5–18); <0.0001
Back pain	−1 (−6 to 1); 0.14	5 (0–8); <0.0001	13 (10–22); <0.0001

We explored these differences further by correlating the differences between expectation and achievement presented in Table 2—which we have described as ‘failure to achieve’ expectations—with both confidence in, and importance of, achieving expectation (Table 3). The rank correlations, though modest, are all positive, and the rank correlations between expectation and importance are statistically significant at all time points, indicating that patients who place higher importance on their ability to achieve expectations tend to experience the largest disappointment or failure to achieve what they expect.

**Table 3:** Rank correlations of failure to achieve expectations for VAS health post-operatively with confidence and importance pre-operatively (*p* value)

Variable	6 weeks	6 months	12 months
Confidence	0.12 (0.07)	0.06 (0.34)	0.09 (0.12)
Importance	0.14 (0.03)	0.17 (0.01)	0.16 (0.01)

### Satisfaction

**Fig. 3:**
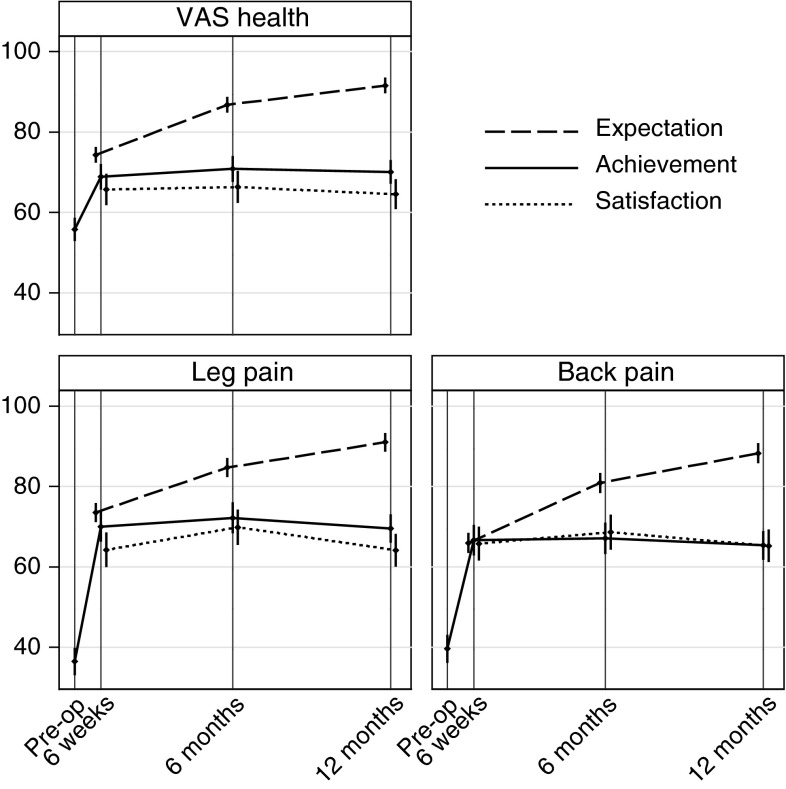
Expectation, achievement and satisfaction after surgery (mean and 95 % confidence intervals for whole group, scored out of 100, VAS pain 100 = no pain, VAS health 100 = ideal health)

Figure 3 depicts mean patient expectation, achievement and satisfaction at 6 weeks, 6 and 12 months post-operatively. There was an expectation that VAS health, back pain and leg pain would improve steadily over the year following surgery, whereas following a substantial initial improvement from their pre-operative state, there was minimal change in achievement after the 6 weeks post-operative assessment. Median scores for satisfaction remained relatively high, thus despite outcomes not matching expectations patients remained relatively satisfied.

Table 4 compares mean differences in satisfaction with improvement between the groups. This table suggests that patients receiving rehabilitation are more satisfied than those not receiving rehabilitation (as all treatment effect estimates are positive), and in contrast those who did not receive a booklet were more satisfied than those who did (as all treatment effect estimates are negative). However, only the effect of rehabilitation on leg pain at 12 months is statistically significant (i.e. the confidence interval for the effect of rehabilitation excludes zero).

**Table 4:** Effects of rehabilitation and booklet interventions on satisfaction with improvement at 6 and 12 months (95 % confidence intervals in parentheses)

Treatment	Variable	6 months	12 months
Rehab vs. no rehab	VAS health	3 (−6 to 12)	4 (−3 to 11)
	Leg pain	8 (−1 to 17)	9 (1 to 17)*
	Back pain	4 (−5 to 14)	3 (−5 to 11)
Booklet vs. no booklet	VAS health	−5 (−14 to 4)	−5 (−12 to 2)
	Leg pain	−6 (−15 to 2)	−6 (−13 to 2)
	Back pain	−6 (−15 to 3)	−6 (−14 to 2)

* Denotes significance at *P* < 0.05 level

Table 5 summarises patient satisfaction with improvement at 6 weeks, 6 and 12 months according to surgical procedure. The satisfaction VAS scores range from 0 (totally dissatisfied) to 100 (very satisfied) with respect to overall health status, leg pain and back pain. Median VAS scores ranged from 66 to 91. Satisfaction tended to be highest at 6 months. We present the differences in satisfaction with improvement between the two types of surgery in Table 6. Patients having discectomy surgery were more satisfied with the outcome in relation to VAS health, back and leg pain (i.e. the confidence interval for the effect of type of surgery excludes zero). As the decompression group was older on average than the discectomy group, we wanted to check that the estimated difference between the different procedures was not due to age, so we also adjusted these estimates for age. Results were largely similar, although the result for back pain at 6 months was not statistically significant (because the estimate was slightly closer to 0 and the confidence interval a little wider).

**Table 5:** Satisfaction with improvement by surgical procedure at 6 weeks, 6 and 12 months, median (quartiles)

Variable	Surgery type	6 weeks	6 months	12 months
Vas health	Discectomy	74 (50, 92)	83 (46, 98)	84 (49, 92)
	Decompression	74 (42, 92)	78 (42, 95)	69 (34, 88)
Leg pain	Discectomy	77 (48, 98)	91 (68, 100)	88 (55, 99)
	Decompression	69 (29, 92)	77 (37, 97)	66 (24, 90)
Back pain	Discectomy	79 (45, 94)	84 (66, 97)	84 (50, 96)
	Decompression	77 (43, 94)	80 (42, 97)	75 (29, 93)

**Table 6:** Difference in satisfaction with improvement between decompression and discectomy surgical procedures at 6 weeks, 6 and 12 months (95 % confidence intervals in parentheses)

Surgery type	Variable	6 weeks	6 months	12 months
Discectomy vs.	VAS health	1 (–7 to 10)	6 (–2 to 14)	10 (3 to 17)*
Decompression	Leg pain	9 (0 to 18)	11 (3 to 19)*	15 (7 to 23)*
	Back pain	2 (–7 to 11)	12 (4 to 20)*	7 (–1 to 15)

* Denotes significance at *P* < 0.05 level

## Discussion

This study explored patients’ expectations of and satisfaction with the surgical outcome of two common spinal interventions: discectomy and decompression. Although the assessment approach used to explore satisfaction and expectation has not been thoroughly validated, they produced some interesting results. Patient expectations of outcome with respect to back and leg pain and health status were elicited prior to surgery and allocation into one of the four arms of the trial, while satisfaction was assessed at each of the key post-operative assessments and as such may be influenced by the group allocation. Consequently, it was observed that patients allocated to receive rehabilitation did express significantly higher satisfaction levels for leg pain improvement, despite the main trial outcome paper suggesting that rehabilitation had little if any impact on functional outcome [24]. Based on global assessment of satisfaction, Cherkin et al. [6] noted a similar finding in the management of chronic low back pain with higher levels of satisfaction in those receiving rehabilitation compared to those allocated to an educational intervention. While these findings may reflect the higher levels of support and personal communication, it is not in line with Kincey et al.’s [17] observation that better informed patients are more satisfied. However, George and Hirsh [9] would argue that in the current study both interventions delivered more information to the patients and that since only satisfaction with symptoms was assessed, the method for delivery of support is less important. It may, however, simply reflect the fact that those receiving rehabilitation felt better supported and cared for. It is also interesting in relation to Ronnberg et al.’s [30] findings that only 46 % of patients were satisfied with the information they were given before surgery. This confirms the importance of providing clear information to patients prior to surgery and ensuring that patients understand this information.

In accordance with past studies, our patient population did expect better outcomes at 1 year than they achieved [12, 21, 26], but contrasts with Yee et al.’s [37] work where 81 % of patient’s expectations of surgery were met. Some work has suggested that the more you expect, the more you will achieve [10, 19, 30, 34], although recent work has refuted this suggesting that it is the discrepancy between expectation and actuality that predicts satisfaction [21]. Our findings suggest that the more important it is for you to achieve a good outcome, the more likely you are to be disappointed. The importance of achieving a good outcome may reflect the patients’ ability to cope with their low back and leg pain and its impact on their lifestyle. This requires further exploration. Mannion et al. [21] speculated that many patients were over-optimistic with regard to the outcome of their surgery, a concept that gained support from Carragee and Cheng [5]. Carragee and Cheng [5] implemented the concept of minimally acceptable outcomes for spinal fusion surgery, but noted that for many patients the minimal acceptable outcome was set very high, and in excess of the current minimum clinically important difference.

De Groot et al. [7] had a slightly different take on the discrepancy between expectation and achievement, preferring to define this as disappointment rather than dissatisfaction. Indeed, this term is perhaps a more pertinent reflection of expectations not being met, particularly if one considers them to be what patients hope for as was suggested by Rao et al. [29]. De Groot et al. [7] goes on to discuss the finding of disappointment in terms of optimism, suggesting that optimists fare better from surgery than pessimists and that perhaps it is harmful to attempt to alter or change optimistic behaviours about outcome. Mannion et al. [21] expressed similar caution on interventions designed to lower patient expectation whilst still noting that having expectations fulfilled was important to the patient. Thus, it appears that this conflict between expectation and outcome may not be as negative as once thought, and that achieving patient expectations is not the Holy Grail. Mannion et al. [20, 22] have recently proposed a new paradigm in outcome assessment and that is the move towards measures of bothersomeness in relation to key presenting symptoms and their persistence following surgery. This measure allows the clinician to gain a perspective of the impact such systems have on a patient’s quality of life and as such may be more meaningful than expectation of outcome.

Similar parallels were noted between expectations and confidence in this study. Overall, the majority of patients were confident in achieving their goals, which fits with De Groot et al.’s [7] model of optimism. As with expectations, importance in achieving outcome did not equate to a better outcome in this study.

It was interesting to note that differences existed in the satisfaction with outcome between the two surgical populations, with discectomy patients clearly having higher satisfaction levels. Few studies have explored the influence of surgical procedure apart from Toyone et al. [34], who also noted higher levels of satisfaction in the discectomy population with little difference in pre-operative expectation between the two groups. It is not clear from either study why this difference occurs; however, in our earlier outcome study [24] it was observed that patients having discectomy did achieve better outcomes than those having decompression which may relate to the underlying physiological process and the differences in age between the two populations.

Finally, despite a disparity between achievement and expectations, the majority of patients did express high levels of satisfaction, 74 % and above. However, it is not clear what impact the study interventions and the continued contact with trial staff and study reviews may have had on this, as clearly this is higher than the levels previously reported [26]. Considering the multi-faceted nature of satisfaction it is not clear what is an acceptable level of patient satisfaction, and perhaps future research considering bothersomeness of symptoms [22] and satisfaction may yield more forthcoming results. However, it is clear from other aspects of this clinical trial that aspects of patient experience could be improved which would impact on satisfaction with their hospital experience, the surgeon’s communication and their waiting times for surgery and consultation [11, 25], all factors identified recently as impacting on patient satisfaction [11].

In summary, the findings of this study confirm previous work that suggests patients’ expectations of surgical outcome exceed their achievement. The importance of a good outcome or indeed the patient’s confidence in achieving a good outcome did not equate to a good result, indeed attaching high importance to a good outcome was correlated with a failure to achieve expectations. However, satisfaction levels remained high, with some indication that the surgical decompression population were less satisfied than the discectomy, and this effect was not due to the different ages of the surgical groups. Further work is required to establish whether unrealistic expectations are detrimental to recovery.

## Appendix: Faster team

### Trial Steering Committee

Professor Jeremy Fairbank

Mrs Caroline Doré

Lord ER Oxburgh

Professor Jennifer Klaber Moffett

Mr Steve Eisenstein

Mr Tim Morris

Professor Alison McGregor

Trial Coordinator—see below

### Trial Management Team

Principle investigator: Alison McGregor

Trial Coordinators: Ania Henley, Jack Kerr, Dr Julia Toschke

Physiotherapy Coordinators: Carla Ashford, Janet Deane, Carol Waugh

### Data Monitoring and Ethics Committee

Professor Sarah Hewlett

Dr Simon Bowman

Dr Martyn Lewis

### Participating Hospitals

Charing Cross Hospital

Guy’s and St Thomas’ Hospital

Heatherwood Hospital

Ravenscourt Park Hospital

Royal Free Hospital

St Mary’s Hospital

Wexham Park Hospital

### Contributing Surgeons

Mr M Akmal

Mr N Anjarwalla

Mr R Bradford

Mr N Dorward

Mr T Ember

Mr D Fahy

Professor S Hughes

Mr J Johnson

Mr K Lam

Mr J Lucas

Mr N Mendoza

Ms M Murphy

Mr D Nandi

Mr J O’Dowd

Mr K O’Neill

Mr D Petersen

Mr C Shieff

Mr L Thorne

Mr C Ulbricht

Professor J van Dellen

### Trial Physiotherapists

Katie De Albuquerque

Nathan Allwork

Hayley Boyle

Ann Bryan

Rachel Freeman

Jenny Heal

Annys Hawkins

Catherine Heathcote

Frances Honniball

Gary Jones

Biljana Kennaway

Gemma Kettle

Anna Kuppuswamy

Claire Marshall

Ann McCarthy

Hannah Mundy

Sandra Noonan

Sara Parker

Camilla Pleydell-Bouverie

Rebecca Portwood

Amy Powel

Adam Royffe

Victoria Salmon

Laura Segar

Alison Sentence

Tom Stenning

Hillary Thompson

Lorraine Tomeldan

Amanda Wall
